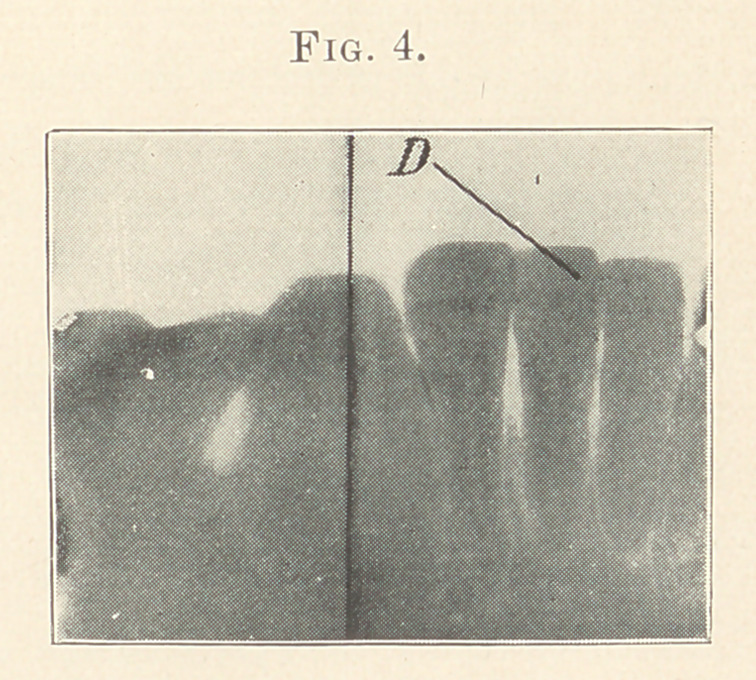# X-Rays

**Published:** 1898-02

**Authors:** Dwight M. Clapp

**Affiliations:** Boston, Mass.


### X-RAYS.

BY DWIGHT M. CLAPP, D.M.D., BOSTON, MASS.

A LITTLE over a year ago, in a talk with Dr. William Rollins, of this city, I became interested in X-rays. Since then I have worked with it more or less constantly, and I give here a very few examples, of the many I have, showing the utility of the discovery to us in the general practice of dentistry.

FIG. 1.

FIG. 2.

FIG. 3.

FIG. 4.

Fig. 1.—Patient twenty-three years of age with temporary cuspid still in place. X-ray shows the permanent cuspid embedded in the jaw, in a nearly horizontal position. The left side of this mouth is almost an exact duplicate of the right.

Fig. 2.—Showing a broken Gates-Glidden drill, which had been forced through the apex of the root of central. Discovered by use of the X-ray, after having remained in the jaw more than a year, causing severe abscess. The light spot around the drill shows considerable breaking down of tissue. The piece of drill removed was three-eighths of an inch long.

Fig. 3.—Showing fracture of inferior central, near the apex of the root, caused by blow from a polo mallet.

The picture was taken on July 21 last, a day or two after the accident. This and several of the adjoining teeth were quite loose, but there was no soreness nor signs of inflammation. A gold splint was made and cemented over the tooth, and worn until about the 1st of November. At this time the teeth were quite firm, showing in a strong light no signs of devitalized pulps nor any other trouble.

Fig. 4.—This was taken on November 11. It gives no trace of the fracture. There appears to be a slight thickening, as though an osseous deposit had taken place. (The negative shows this better than the reproduction.) Has there been a union of the fracture?

The patient, a young and vigorous man, has gone to the Hudson Bay country for a winter's sport, hunting big game. When he returns in the spring I hope to get more definite information regarding the condition of the tooth.

I use a double-plate static machine, driven by a sixth horsepower motor. The power to drive the motor is taken from the street current.

The exposures are from two to three and one-half minutes, at a distance of from eight to ten inches. The films are one and five-eighths by one and one-quarter inches, wrapped in three thicknesses of non-actinic paper.

## Figures and Tables

**Fig. 1. f1:**
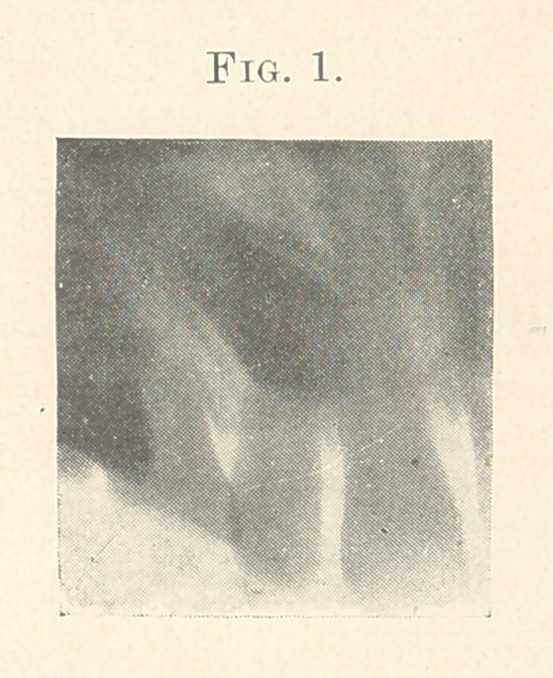


**Fig. 2. f2:**
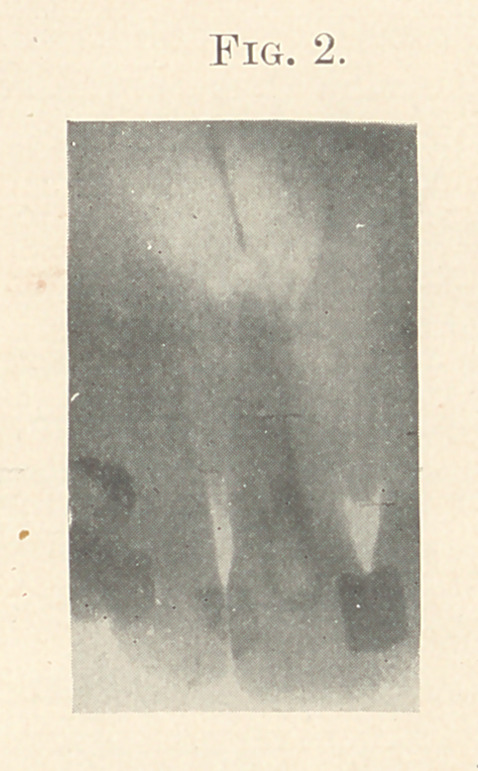


**Fig. 3. f3:**
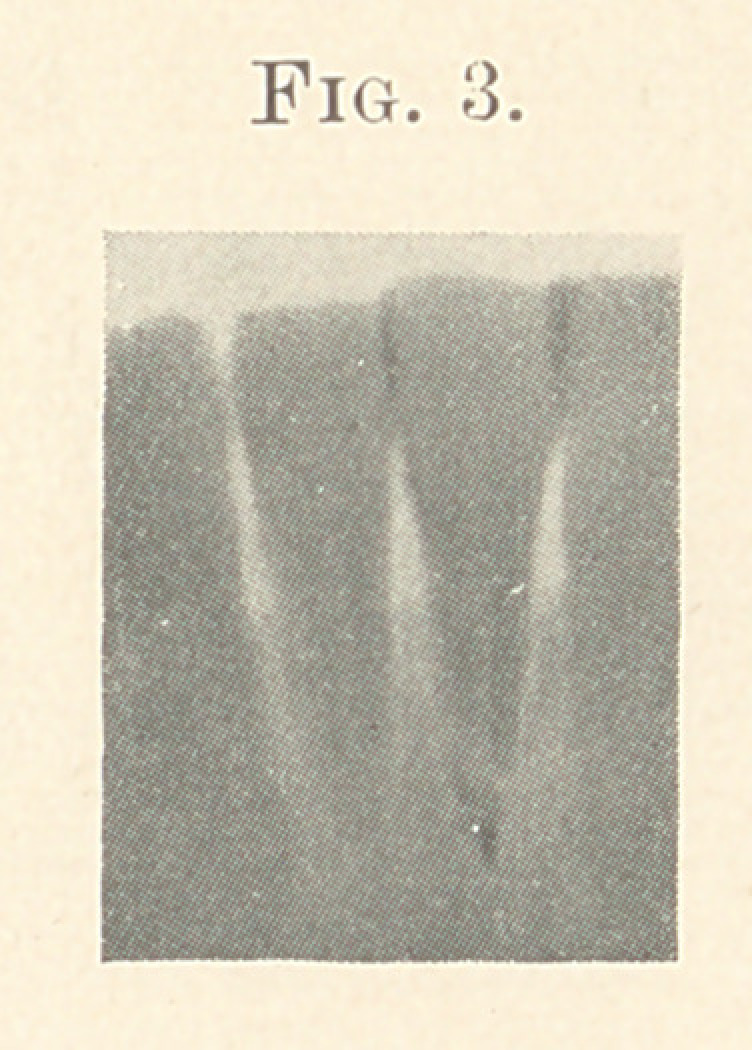


**Fig. 4. f4:**